# A first principles method to determine speciation of carbonates in supercritical water

**DOI:** 10.1038/s41467-019-14248-1

**Published:** 2020-01-21

**Authors:** Ding Pan, Giulia Galli

**Affiliations:** 1 Department of Physics and Department of Chemistry, Hong Kong University of Science and Technology, Hong Kong, China; 2 HKUST Fok Ying Tung Research Institute, Guangzhou, China; 3 Pritzker School of Molecular Engineering, The University of Chicago, Chicago, IL 60637 USA; 4 Department of Chemistry, The University of Chicago, Chicago, IL 60637 USA; 5 Materials Science Division and Center for Molecular Engineering, Argonne National Laboratory, Argonne, IL 60439 USA

**Keywords:** Raman spectroscopy, Density functional theory, Molecular dynamics

## Abstract

The determination of the speciation of ions and molecules in supercritical aqueous fluids under pressure is critical to understanding their mass transport in the Earth’s interior. Unfortunately, there is no experimental technique yet available to directly characterize species dissolved in water at extreme conditions. Here we present a strategy, based on first-principles simulations, to determine ratios of Raman scattering cross-sections of aqueous species under extreme conditions, thus providing a key quantity that can be used, in conjunction with Raman measurements, to predict chemical speciation in aqueous fluids. Due to the importance of the Earth’s carbon cycle, we focus on carbonate and bicarbonate ions. Our calculations up to 11 GPa and 1000 K indicate a higher concentration of bicarbonates in water than previously considered at conditions relevant to the Earth’s upper mantle, with important implications for the transport of carbon in aqueous fluids in the Earth’s interior.

## Abstract (web summary)

The determination of the speciation of ions and molecules in supercritical aqueous fluids under pressure is key to understanding their mass transport in the Earth’s interior. Here the authors present a strategy based on ab-initio molecular dynamics to determine the speciation of carbonates in aqueous fluids.

## Introduction

The carbon cycle in the atmosphere and biosphere has important consequences for the Earth’s climate and thus is a topic of intense investigation in many fields of science, including chemistry, environmental science, and geoscience^1,2^. On the Earth, carbon is not only found in near-surface reservoirs, but it is also stored in the interior. In fact, >90% of the Earth’s carbon is buried in the Earth’s interior^3^, and the carbon cycle in the deep Earth greatly influences the carbon budget near the surface, with a substantial impact on global climate change and human energy consumption^4^. After decades of study, our knowledge of the global carbon cycle in the atmosphere, oceans, and the shallow crust has improved considerably; however, relatively little is known about deep carbon reservoirs^4,5^. Water, an important component of geofluids in the Earth’s crust and mantle, may carry a significant amount of carbon, but its dissolved forms in aqueous geofluids are still poorly known, thus limiting our overall understanding of mass transport of carbon in the Earth. This lack of knowledge is due to experimental difficulties in measuring which species are present in C–H–O fluids at extreme conditions, as well as to the use, on the theoretical side, of simplified models^6,7^. For example, C–H–O fluids at the conditions of the Earth’s interior have often been modeled as mixtures of neutral gas molecules (e.g., refs. ^8,9^). However, recent studies pointed out the importance of chemical reactions occurring in deep aqueous fluids, involving the presence of ionic products^10–12^; for example, using first-principles simulations we recently suggested that the molecular CO_2_(aq) species may completely convert into solvated carbonate and bicarbonate ions in water-rich fluids at high temperature and pressure^13^.

At present, the most used experimental tool to investigate chemical speciation in C–H–O fluids under extreme conditions is Raman spectroscopy^14^. The vibrational frequencies obtained from Raman spectra are used as fingerprints to identify molecules or ions, and in principle the concentration of chemical species may be obtained from vibrational peak intensities. Indeed, the concentration of a given species (*C*_*i*_) is proportional to the corresponding Raman intensity *I*_*i*_ = *γ*_*i*_*C*_*i*_, where *γ*_*i*_ is the Raman scattering cross section. However, under extreme conditions, the variation of *γ*_*i*_ with pressure (P) and temperature (T) is unknown; hence the concentration of chemical species may not be inferred from Raman peak intensities. Several estimates of the dependence of the Raman cross-sections of aqueous solutions at high P (HP) and high T (HT) have been reported in the literature; these estimates are not only controversial but in some cases they provide contradicting results. Based on geochemical models, Frantz predicted that the *γ* ratio between carbonate and bicarbonate ions in aqueous fluids, $\frac{{\gamma }_{{\rm{carb}}}}{{\gamma }_{{\rm{bicarb}}}}$, decreased by 75% with increasing temperature from 523 to 823 K, and did not vary with increasing pressure^15^. However, Schmidt suggested that this ratio changed by no >10% from 296 to 873 K and up to 1 GPa, based on the comparison of the Raman bands of (bi)carbonate ions with those of similar ion pairs, sulfate and hydrogen sulfate ions, under similar conditions^16^.

The lack of knowledge of the *P*–*T* dependence of Raman scattering cross-sections poses serious limitations to the use of Raman spectroscopy to determine chemical speciation of C–H–O fluids in the deep Earth, and as a consequence to our understanding of the deep carbon cycle, since at present Raman spectroscopy has been the technique of choice to investigate these liquids. Indeed the use of other spectroscopic methods, e.g., infrared^17^ or nuclear magnetic resonance^18^, is challenging under extreme *P*–*T* conditions and it is not known how to derive the concentrations of dissolved species solely from measured spectroscopic data.

**Fig. 1:**
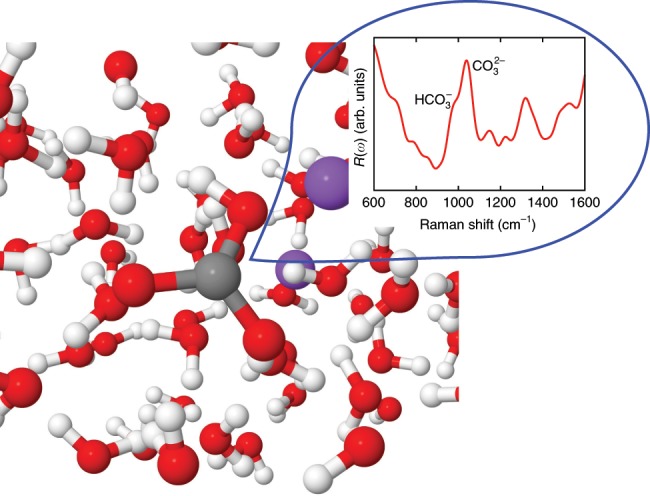
A snapshot of the sodium carbonate solution at  ~10 GPa and 1000 K. The configuration was selected from first-principles molecular dynamics simulations trajectories (see text). The inset shows the calculated unpolarized Raman spectrum of the solution (red line).

Here, we present a strategy, based on first-principles simulations at extreme conditions, to determine the ratios of the Raman scattering cross-sections, thus providing a key quantity that can be used, in conjunction with Raman measurements, to predict speciation in aqueous fluids. In particular we present the results of first-principles molecular dynamics (FPMD) simulations of Na_2_CO_3_ and NaDCO_3_ aqueous solutions at HP-HT conditions (see Fig. 1). Our simulations permit the determination of the concentration of species in the solution^13^, as well as of their Raman spectra, including peak positions and intensities. This combined information can then be used to obtain Raman cross-sections. To the best of our knowledge, our study represents the first determination of Raman cross section ratios between aqueous carbonate and bicarbonate ions up to  ~11 GPa and 1000 K and hence the first determination of speciation in these aqueous fluids. We find that this ratio decreases with increasing pressure, while it increases with increasing temperature, at variance from conclusions reached using geochemical models. Our results point at the presence of a higher concentration of bicarbonate ions at extreme conditions than previously thought, with important consequences for carbon transport in the Earth.

## Results

### Raman spectra at ambient density

**Fig. 2:**
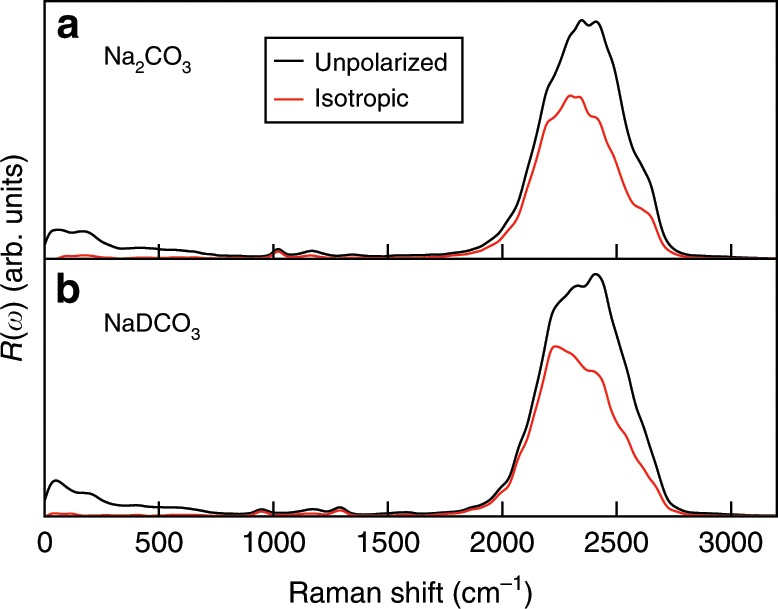
Raman spectra of 0.9 mol L^−1^ solutions at ambient density and 380 K. Calculated unpolarized (black line) and isotropic (red line) spectra of the sodium carbonate and deuterated sodium bicarbonate solutions are shown in the upper and lower panels, respectively.

**Fig. 3:**
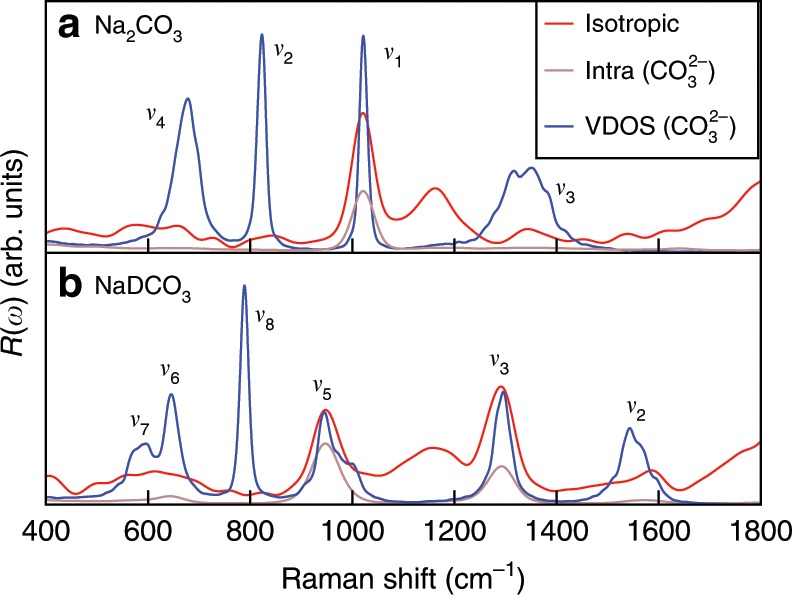
Zoomed-in Raman spectra of 0.9 mol L^−1^ solutions at ambient density and 380 K. Calculated spectra of the sodium carbonate and deuterated sodium bicarbonate solutions are shown in the upper and lower panels, respectively. The isotropic Raman spectra (red line), the intra-molecular Raman spectra (brown line), and the vibrational density of states (VDOS) (blue line) of CO${}_{3}^{2-}$ are compared.

Figure 2 shows the computed unpolarized and isotropic Raman spectra of 0.9 mol L^−1^ Na_2_CO_3_ and NaDCO_3_ solutions at ambient density and 380 K. We considered 380 instead of 300 K to better reproduce the experimental radial distribution functions and diffusion constant of water at ambient conditions with the functional adopted here (PBE^19^). The solute concentration was set to a value similar to that determined experimentally^15^. The Raman spectra were obtained using FPMD simulations, which account for an-harmonic effects (see Methods), critical to describe fluids. At these conditions, we found that <1% of carbonate and bicarbonate ions react in the Na_2_CO_3_ and NaDCO_3_ solutions. The broad band at  ~2400 cm^−1^ shown in the figure originates from the water stretching mode^20^. The unpolarized and isotropic spectra show similar features, except at low frequency, in the range of 0~700 cm^−1^, where the baselines of the isotropic spectra are closer to zero than those of the unpolarized ones, consistent with experimental observations^21^. Following a procedure commonly adopted in experiments^21^, we used isotropic spectra to obtain Raman peak intensities. We first analyze the weak peaks between 400 and 1800 cm^−1^ (see Fig. 3). We assigned those peaks by using the computed vibrational densities of states (VDOS) and the intra-molecular Raman spectrum of the CO${}_{3}^{2-}$ species. The VDOS of CO${}_{3}^{2-}$ was obtained by Fourier transforming the velocity–velocity auto-correlation function:

1 $${I}_{\mathrm{{C{O}}}_{3}^{2-}}^{{\mathrm{VDOS}}}=\int dt{{\rm{e}}}^{-{\rm{i}}\omega t}\frac{\sum _{i\in {\mathrm{C{O}}}_{3}^{2-}}\langle {{\bf{v}}}_{i}(0)\cdot {{\bf{v}}}_{i}(t)\rangle }{\sum _{i\in {\mathrm{C{O}}}_{3}^{2-}}\langle {{\bf{v}}}_{i}(0)\cdot {{\bf{v}}}_{i}(0)\rangle },$$

where *t* is the correlation time, *ω* is the frequency, and **v**_*i*_ is the velocity of the *i*th atom in the CO${}_{3}^{2-}$ unit. The intra-molecular Raman spectra of CO${}_{3}^{2-}$ were computed using the auto-correlation functions of the effective polarizabilities of CO${}_{3}^{2-}$, ${\alpha }_{\mathrm{CO}_{3}^{2-}}$:

2 $${\mu }_{\mathrm{{C{O}}}_{3}^{2-}}={\alpha }_{{\mathrm{C{O}}}_{3}^{2-}}{\bf{E}}.$$

In Eq. (2), ${\mu }_{{\mathrm{C{O}}}_{3}^{2-}}$ is the induced polarization of CO${}_{3}^{2-}$ (computed from the centers of maximally localized Wannier functions) and **E** is the macroscopic electric field (see Supplementary Methods).

The carbonate ion has four normal vibrational modes^22^, clearly visible in the VDOS of CO${}_{3}^{2-}$: the *ν*_1_ symmetric stretching at 1021 cm^−1^, the *ν*_2_ out-of-plane deformation at 823 cm^−1^, the *ν*_3_ asymmetric stretching at ~1350 cm^−1^, and the the *ν*_4_ in-plane deformation at 678 cm^−1^. In the full and intra-molecular Raman spectra, the *ν*_1_ peak is the most intense, and it has thus been used as the characteristic Raman signature of carbonate ions. The bicarbonate ion has 9 normal vibrational modes^22^. In Fig. 3, the VDOS corresponding to the bicarbonate ion HCO${}_{3}^{-}$ show at least six vibrational modes: the *ν*_7_(OD) and *ν*_6_(CO) bendings at ~596 cm^−1^ and 645 cm^−1^, respectively, the *ν*_8_(CO_3_) out-of-plane deformation at 788 cm^−1^, the *ν*_5_(C-OD) symmetric stretching at 945 cm^−1^, the *ν*_3_(CO) symmetric stretching at 1296 cm^−1^, and the *ν*_2_(CO) asymmetric stretching at  ~1542 cm^−1^. In the full and intra-molecular Raman spectra, only the *ν*_5_(C-OD) and *ν*_3_(CO) modes can be easily identified. In both Raman spectra of the carbonate and bicarbonate solutions we found a broad band at ~1150 cm^–1^, which is not present in the VDOS and intra-molecular Raman spectra of CO${}_{3}^{2-}$. A similar band can be found in the Raman spectrum of pure water at ambient conditions^20^, and hence it was assigned to the bending mode of liquid water. We note that the calculated Raman frequencies are systematically lower than the experimental ones by  ~40 cm^−1^ (ref. ^23^); this underestimate is ascribed to the exchange-correlation functional chosen in our simulations (a gradient-corrected functional: PBE^19^). It is well known that generalized gradient approximations underestimate the vibrational frequencies of covalent bonds^24^ and we do not consider this underestimate a serious drawback of our simulations, which we are using to assign species and understand trends, rather than to obtain exact values of vibrational frequencies. Importantly, our calculated unpolarized and isotropic Raman spectra are overall in good agreement with experimental ones. We also note that quite a few modes are present in the VDOS but not in the computed Raman spectra, indicating that the Raman selection rules included in our calculations are correctly reproducing experimental findings.

### Raman spectra at high pressure and temperature

**Fig. 4:**
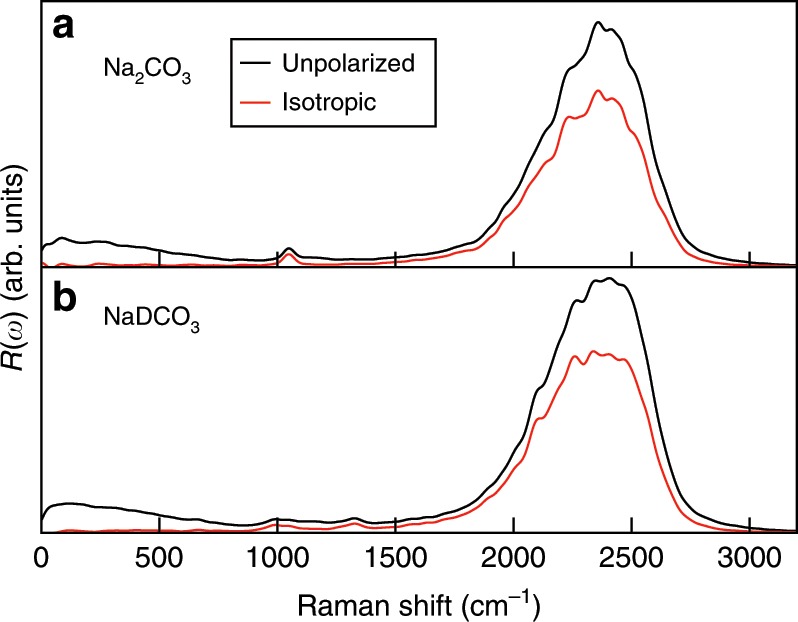
Raman spectra of 0.9 molality solutions at  ~9 GPa and 600 K. Calculated unpolarized (black line) and isotropic (red line) spectra of the sodium carbonate and deuterated sodium bicarbonate solutions are shown in the upper and lower panels, respectively.

**Fig. 5:**
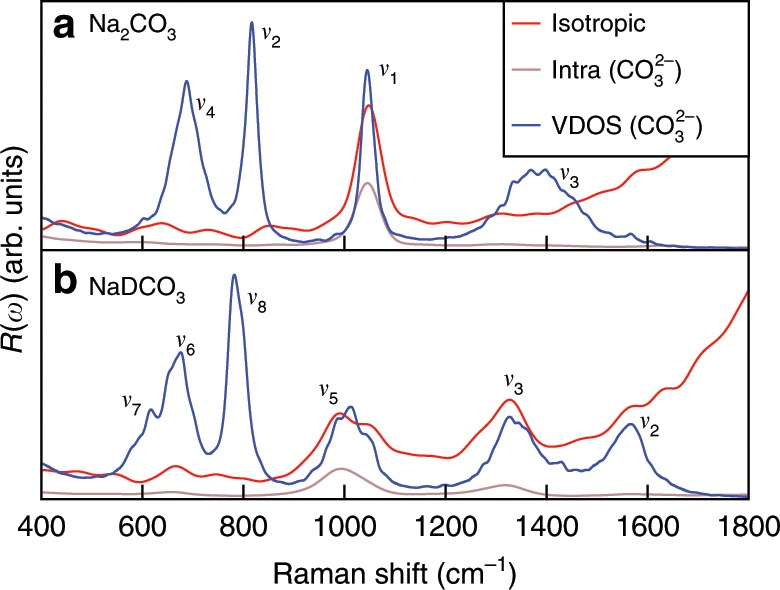
Zoomed-in Raman spectra of 0.9 molality solutions at  ~9 GPa and 600 K. Calculated spectra of the sodium carbonate and deuterated sodium bicarbonate solutions are shown in the upper and lower panels, respectively. The isotropic Raman spectra (red line), the intra-molecular Raman spectra (brown line), and the vibrational density of states (VDOS) (blue line) of CO${}_{3}^{2-}$ are compared.

Having interpreted the low pressure and temperature spectra, we turned to solutions under extreme conditions. We increased the pressure of our simulations to  ~9 GPa and the temperature to 600 K. At these conditions we observed the conversion between carbonate and bicarbonate ions. In the molecular dynamics (MD) simulation of 0.9 m (molality) Na_2_CO_3_, the mole percent of DCO${}_{3}^{-}$ per total carbon species is 8.5% and the remaining carbon species are CO${}_{3}^{2-}$. In the 0.9 m NaDCO_3_ solution, we found instead 18.5% CO${}_{3}^{2-}$, 81.3% DCO${}_{3}^{-}$, and 0.2% D_2_CO_3_. The mole percents were obtained from MD trajectories based on the molecular geometry of the solutes (see the Methods section in ref. ^13^). Figure 4 shows the full Raman spectra of these two solutions, and Fig. 5 shows a specific frequency region. The calculated spectra are overall similar to those obtained at ambient conditions, but the vibrational bands are wider and the Raman active modes, *ν*_1_ of carbonate ions, *ν*_5_(C-OD) and *ν*_3_(CO) of bicarbonate ions, are all shifted to higher frequencies relative to ambient conditions. It has been experimentally reported that the Raman frequencies of the *ν*_1_ and *ν*_5_(C-OH) modes both increase with increasing pressure at a rate of ~6.5 and ~14 cm^−1^ per GPa, respectively, and decrease with increasing temperature at a rate of ~−0.035 and ~−0.096 cm^−1 ^per K, respectively^16^; hence our results, obtained at  ~9  GPa and 600 K, indicate that the pressure effect outweighs the temperature one and all major Raman peaks are blue-shifted compared to those at ambient conditions.

**Fig. 6:**
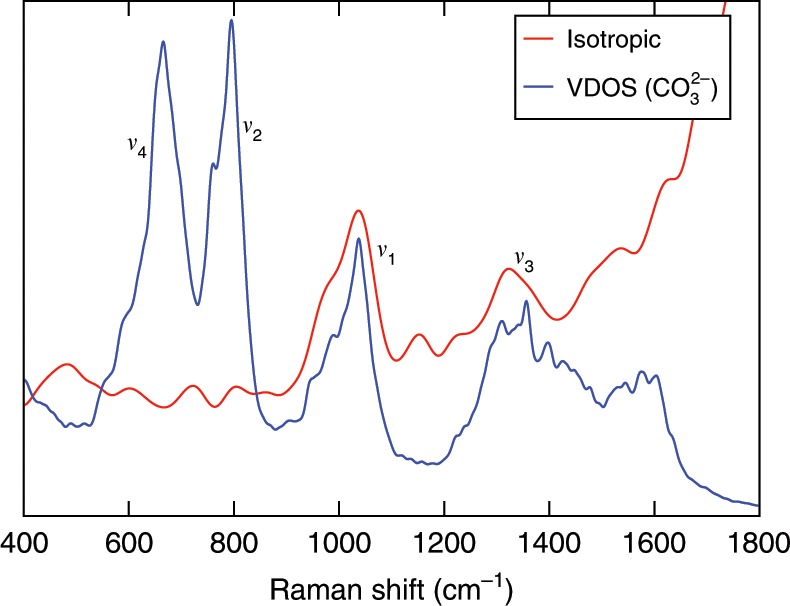
Raman spectra of the 0.9 molality sodium carbonate solution at  ~11 GPa and 1000 K. The isotropic Raman spectrum of this solution (red line) and the vibrational density of states (VDOS) (blue line) of CO${}_{3}^{2-}$ are compared.

Upon further increase of the pressure of the 0.9 m Na_2_CO_3_ solution to  ~11 GPa and the temperature to 1000 K, we found 40.6% CO${}_{3}^{2-}$, 57.7% DCO${}_{3}^{-}$, and 1.6% D_2_CO_3_. At these conditions, the carbonate and bicarbonate ions are rapidly converting into each other, and their lifetime is estimated to be on the sub-picosecond time scale, as reported in our previous study^13^. Figure 6 shows the isotropic Raman spectrum and VDOS of this solution. The Raman peaks are much broadened, due to the decreased lifetime of ions in the solution. Again, with the help of the computed VDOS and the intra-molecular Raman spectrum of CO${}_{3}^{2-}$, we identified the *ν*_1_ mode of carbonate ions and the *ν*_3_(CO) mode of bicarbonate ions. The *ν*_1_ band exhibits a shoulder, which is primarily assigned to the *ν*_5_(C-OD) mode of bicarbonate ions. The Raman signal of ion pairs NaCO${}_{3}^{-}$ is expected to be between the *ν*_1_ and *ν*_5_(C-OD) bands^16^.

We note that while our calculations for the bicarbonate ion show the presence of two Raman bands (see Fig. 5), *ν*_5_(C-OD) and *ν*_3_(CO), in high-pressure experiments conducted in diamond anvil cells, only the *ν*_5_(C-OD) mode is detected. The reason is that the Raman band of the diamond window is at 1332 cm^−1^, i.e., very close to the Raman frequency of the *ν*_3_(CO) mode, and it becomes broader at high *P* and *T*^25^ and thus almost indistinguishable from the *ν*_3_(CO) mode.

## Discussion

Having assigned all peaks in our computed spectra, and having determined the corresponding concentrations of ionic species from the MD trajectories, we are now in a position to directly compute *γ* from the Raman peak intensities and the concentrations of species. As mentioned in the introduction, *γ* is precisely the information not directly available in experiments at high *P* and *T*, and required to determine speciation from measured Raman spectra. We recall that at ambient conditions, and at 600 K and  ~9 GPa, we calculated the Raman spectra of Na_2_CO_3_ and NaDCO_3_ solutions in separate simulations; therefore before proceeding to the determination of the *γ* ratio, the two spectra obtained in independent simulations need to be aligned. Since we simulated the Na_2_CO_3_ and NaDCO_3_ solutions with the same number of water molecules, we can use the water stretching band as our calibration to align the spectra. We found that the calculated *γ* ratio decreases with increasing Gaussian broadening width used to smooth the computed spectra; hence for comparison purposes, we adopted the same width in all the Raman spectra. The *γ*-ratio, $\frac{{\gamma }_{\mathrm{carb}}}{{\gamma }_{\mathrm{bicarb}}}$, obtained from our simulations at ambient conditions is 1.57, which is comparable to the experimental value 1.46 reported by Rudolph et al.^21^, who also observed that the *γ*-ratio did not change up to 492 K. Both the experimental and calculated *γ*-ratios suggest that the Raman scattering cross section of carbonate ions is larger than that of bicarbonate ions, consistent with the expectation that more symmetric species, such as the carbonate ion, have a larger *γ* for the symmetric stretching modes. At 600 K and  ~9 GPa, the computed *γ* ratio is 1.60, essentially the same as that found at ambient conditions. At 1000 K and  ~11 GPa, the determination of the *γ* ratio is much more challenging, because the Raman signal of bicarbonate ions is hidden in the shoulder of the Raman peak of carbonate ions at 1037 cm^−1^. In this case no peak alignment was needed, because both ions were simulated in the same solution. We found a weak peak at 989 cm^−1^ in the VDOS of CO${}_{3}^{2-}$, which may be assigned to the *ν*_5_(C-OD) mode of bicarbonate ions. Using the Raman intensities at 989 cm^−1^ and 1037 cm^−1^, we calculated the *γ* ratio to be 1.81, which is larger than that at ambient conditions, and at 600 K and  ~9 GPa.

**Fig. 7:**
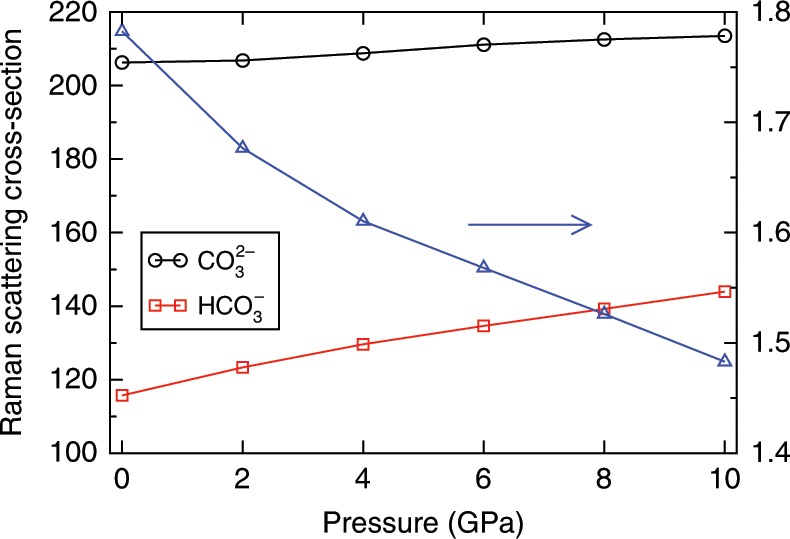
Raman scattering cross-sections of carbonate and bicarbonate ions. We present results for the *δ*-Na_2_CO_3_ (black line) and NaHCO_3_ (red line) crystals as a function of pressure. The cross section ratios between these two ions, i.e., the *γ* ratio ($\frac{{\gamma }_{{\rm{carb}}}}{{\gamma }_{{\rm{bicarb}}}}$), are shown as blue triangles.

To understand how pressure and temperature separately affect the value of *γ*, we computed the Raman spectra of the *δ*-Na_2_CO_3_ and NaHCO_3_ crystals up to 10 GPa and at 0 K in Fig. 7. The space groups of these two crystals are *C*2∕*m*^26^ and *P*12_1_∕*c*1^27^, respectively. We compressed the crystals without allowing for structural phase transitions. Figure 7 shows that both *γ*_carb_ and *γ*_bicarb_ increase with increasing pressure, but *γ*_bicarb_ increases faster than *γ*_carb_, so the *γ* ratio decreases by 17% from 0 to 10 GPa. Our FPMD simulations showed that the *γ* ratio is approximately constant in going from ambient conditions to  ~9 GPa and 600 K; this result, together with the one obtained for the solids under pressure, indicates that the *γ* ratio increases with increasing temperature. If we assume that this ratio linearly depends separately on temperature and pressure, then its value may be obtained from the following relation:

3 $$\frac{{\gamma }_{{\rm{carb}}}}{{\gamma }_{{\rm{bicarb}}}}(P,T)=\frac{{\gamma }_{{\rm{carb}}}}{{\gamma }_{{\rm{bicarb}}}}({P}_{0},{T}_{0})+\alpha (P-{P}_{0})+\beta (T-{T}_{0}),$$

where *α* is the decrease rate with pressure and *β* is the increase rate with temperature. Using the *γ* ratios in Fig. 7 and at  ~9 GPa and 600 K, we can calculate *α* as −2.99 × 10^−2^ GPa^−1^ and *β* as 9.84 × 10^−4 ^K^−1^. To test the validity of Eq. (3), we used the *α*- and *β*-values obtained above to calculate the *γ* ratio at  ~11 GPa and 1000 K, which we also have computed directly from FP simulations: we obtained a value of 1.93, about 7% larger than the one directly obtained from the Raman spectrum in Fig. 6 (1.81). This agreement is not perfect but satisfactory and importantly we found the same trend relative to ambient conditions from our simulations and using Eq. (3).

Our calculation shows that the *γ*-ratio increases with increasing temperature, but decreases with increasing pressure; these results differ from those obtained from geochemical models. Using the concentrations calculated from geochemical models, Frantz predicted that the *γ*-ratio decreased by 75% with increasing temperature from 523 to 823 K, and did not change with increasing pressure^15^. However, Schmidt reported that the *γ-*ratio estimated at ambient conditions from Frantz’s work (0.98) is much smaller than the experimental value (1.46), casting doubts on the generality of Frantz’s model^16^. In addition, at 873 K the *γ-*ratio given by Frantz is only 0.003, indicating that the Raman bands of CO${}_{3}^{2-}$ are extremely weak and almost impossible to detect, contrary to what observed experimentally^16^. Hence, it is likely that the carbon species concentrations given by the geochemical models in Frantz’s work are not accurate. According to Rudolph’s Raman studies, the *γ* ratio changes little from 296 to 492 K^21^. Furthermore, Schmidt suspected that this ratio should not change >10% from 492 to 873 K and up to 1 GPa, but according to Eq. (3), this ratio should increase by 22% in this *P*–*T* range. At high temperatures, underestimating the *γ* ratio leads to an underestimate of the concentration of bicarbonate ions. In our previous study of the Na_2_CO_3_ solutions at 0.2 GPa and 823 K, the mole percent of HCO${}_{3}^{-}$ obtained by Raman spectra and the *γ* ratio at ambient conditions is 68%, which is smaller than the mole percent obtained by the FPMD simulations (73–82%), showing that the concentration of bicarbonate ions is indeed underestimated if we assume that the *γ* ratio does not change with pressure and temperature.

In summary, we presented a strategy to determine the speciation of carbonates in aqueous fluids under pressure, which combines theoretical and experimental spectroscopies. We considered conditions relevant to the Earth’s upper mantle, though our first-principles calculations can be conducted under a variety of different conditions and for numerous other ions and molecules in aqueous environments. In particular we showed how to compute, from first-principles, Raman scattering cross-sections, which are not yet known experimentally, at high pressure and high temperature. The information on cross-sections, together with that of measured Raman intensities, can then be used to obtain the speciation of aqueous carbonates, an essential prerequisite to understand the chemistry of carbon in water at extreme conditions.

Interestingly, we found that the ratio of the Raman scattering cross-sections of carbonate and bicarbonate ions in water, $\frac{{\gamma }_{{\rm{carb}}}}{{\gamma }_{{\rm{bicarb}}}}$, decreases with increasing pressure, while it increases with increasing temperature, up to  ~10 GPa and 1000 K. Our results differ from those reported previously based on geochemical models, showing either a decrease or a constant *γ* ratio under high temperature. These discrepancies are not surprising as the C–H–O fluids were modeled assuming neutral gas species under extreme *P*–*T* conditions, while many recent studies, including our simulations, show reactions between these gas species in aqueous environments. The results obtained here for the *γ* ratio imply a higher concentration of bicarbonate ions than previously considered, with important implications for the transport of carbon in aqueous fluids in the Earth interior.

## Methods

### First-principles molecular dynamics

We performed first-principles MD simulations using the Qbox code (http://qboxcode.org)^28^. The time step was 0.24 fs. We used the PBE exchange-correlation functional^19^ and Hamann-Schluter-Chiang-Vanderbilt norm-conserving pseudopotentials^29,30^ with a plane-wave kinetic energy cutoff of 85 Ry. When calculating pressure, the cutoff was increased to 220 Ry. There are 62 water molecules and one Na_2_CO_3_ or NaDCO_3_ molecule in the cubic simulation box with periodic boundary conditions. We used deuterium instead of hydrogen so as to enable the use of a larger time step in MD simulations. Temperature was controlled by the Bussi-Donadio-Parrinello thermostat^31^, and the time scale of the response of the thermostat (*τ*) is 24.2 fs.

### First-principles Raman spectroscopy

The Raman spectra were calculated by the auto-correlation functions of electronic polarizabilities^20^. The isotropic and anisotropic Raman spectra were obtained respectively by

4 $${R}_{{\rm{iso}}}(\omega )\propto \frac{\hslash \omega }{{k}_{B}T}\int {dt{\rm{e}}}^{-{\rm{i}}\omega t}\langle \bar{\alpha }(0)\bar{\alpha }(t)\rangle$$

5 $${R}_{{\rm{aniso}}}(\omega )\propto \frac{\hslash \omega }{{k}_{B}T}\int {dt{\rm{e}}}^{-{\rm{i}}\omega t}\left\langle \frac{2}{15}{\rm{Tr}}\beta (0)\beta (t)\right\rangle ,$$

where *ω* is the frequency, *k*_*B*_ is the Boltzmann constant, *t* is the correlation time, and Tr is the trace matrix operator, $\bar{\alpha }$ and *β* are the isotropic and anisotropic components of the polarizability tensor *α*: $\bar{\alpha }=\frac{1}{3}{\rm{Tr}}\alpha$ and $\beta =\alpha -\bar{\alpha }{\bf{I}}$, where **I** is the identity tensor. The linear combination of these two spectra gives the unpolarized Raman spectra: ${R}_{{\rm{unpol}}}={R}_{{\rm{iso}}}+\frac{7}{4}{R}_{{\rm{aniso}}}$. The polarizability tensors were calculated by density functional perturbation theory^32^ every 15 MD steps. The length of each MD trajectory used in the auto-correlation calculations is between 210 and 280 ps. A Gaussian broadening is used to smooth spectra, whose full width at half maximum (FWHM) is 45 cm^−1^.

We note that in our MD simulations, nuclei were treated as classical particles; the comparisons between intensities reported for the vibrational spectra of water with classical and quantum dynamics at ambient conditions do not show any qualitative difference and relatively small quantitative differences for the intensities, with blue-shifted frequencies of the order 60–120 cm^−1^ in the case of classical dynamics^33,34^. At elevated temperatures, we expect the small differences observed at ambient conditions to diminish.

## Supplementary information

Supplementary Information

## Data Availability

The data that support this study are available upon request from the authors.

## Code availability

Qbox is a free and open source code available at http://qboxcode.org. Data processing scripts are available upon request from the authors.
