# Supplementary material for: A first principles method to determine speciation of carbonates in supercritical water
Source: Nat Commun. 2020 Jan 21;11:421. doi: 10.1038/s41467-019-14248-1 (PMC6972934; doi:10.1038/s41467-019-14248-1)
Supplement: Supplementary file 1 — Supplementary Information [file 41467_2019_14248_MOESM1_ESM.pdf]

Supplementary Information for "A first principles method to determine speciation of carbonates in supercritical water"

Ding Pan[1,2,*] and Giulia Galli[3,4,5]

[1]*Department of Physics and Department of Chemistry, Hong Kong University of Science and Technology, Hong Kong, China*

[2]*HKUST Fok Ying Tung Research Institute, Guangzhou, China*

[3]*Pritzker School of Molecular Engineering, The University of Chicago, Chicago, Illinois 60637, USA*

[4]*Department of Chemistry, The University of Chicago, Chicago, Illinois 60637, USA*

[5]*Materials Science Division and Center for Molecular Engineering, Argonne National Laboratory, Argonne, Illinois 60439, USA*

SUPPLEMENTARY METHODS

Electronic polarizability from linear response theory

The Kohn-Sham equations of density functional theory read [1]:

$$H_{\mathrm{KS}} \left|\varphi_v\right\rangle = \varepsilon_v \left|\varphi_v\right\rangle , \tag{1}$$

where H_{KS} is the Kohn-Sham one-electron Hamiltonian and φ_v is the vth occupied Kohn-Sham orbital with eigenvalue ε_v. Within linear response and density functional perturbation theory [2], the response to an external perturbation may be written using the Sternheimer equation [3, 4]:

$$(H_{\mathrm{KS}} - \varepsilon_v)\hat{P}_c \left|\Delta\varphi_v\right\rangle = -\hat{P}_c \Delta V_{\mathrm{KS}} \left|\varphi_v\right\rangle , \tag{2}$$

where $\hat{P}_c$ is the projector onto the unoccupied Kohn-Sham orbitals: $\hat{P}_c = \sum_{c=1}^{+\infty} \left|\varphi_c\right\rangle \left\langle\varphi_c\right| = \mathbf{1} - \sum_{v=1}^{N_{\mathrm{orb}}} \left|\varphi_v\right\rangle \left\langle\varphi_v\right|$, N_{orb} is the number of the occupied Kohn-Sham orbitals, and ΔV_{ks} is the perturbation to the Kohn-Sham potential. If the perturbation is caused by a macroscopic electric field $\mathbf{E}$, the perturbation potential ΔV_{ks} is written as

$$\Delta V_{\mathrm{KS}} = \mathrm{e}\mathbf{E}\cdot\mathbf{r} + \Delta V_{\mathrm{H}} + \Delta V_{\mathrm{xc}}, \tag{3}$$

where e is the elementary charge, $\mathbf{r}$ is the position operator, and ΔV_{H} and ΔV_{xc} are the perturbations to the Hartree and exchange-correlation potentials, respectively. After solving Supplementary Equation (2), we can compute the change in electronic density:

$$\Delta\rho = 2\sum_{v=1}^{N_{\mathrm{orb}}} \varphi_v^*(\mathbf{r})\Delta\varphi_v(\mathbf{r}) + c.c. \tag{4}$$

Note that ΔV_{ks} also depends on $\Delta\rho$, so Supplementary Equation (2) has to be solved self-consistently.

The induced dipole moment of the system under the action of a perturbing field is given by:

$$\mathbf{M} = -\mathrm{e}\int_{\Omega} \mathbf{r}\Delta\rho d\mathbf{r}, \tag{5}$$

where Ω is the volume of the system. The polarizability tensor α is defined as:

$$\mathbf{M} = \alpha \mathbf{E} \tag{6}$$

Within periodic boundary conditions, the position operator $\mathbf{r}$ is ill defined, and we use the momentum operator $\mathbf{p}$ instead, to compute matrix elements:

$$\langle \varphi_i | \mathbf{r} | \varphi_j \rangle = \frac{\langle \varphi_i | -\frac{\mathrm{i}\hbar}{m_e}\mathbf{p} + [V_{\mathrm{KS}}, \mathbf{r}] | \varphi_j \rangle}{\varepsilon_i - \varepsilon_j}, i \neq j \tag{7}$$

where $\hbar$ is the reduced Planck constant and m_{e} is the electron mass. The commutator in Supplementary Equation (7) is needed when using nonlocal pseudopotentials to obtain the effective Kohn-Sham potential [5].

We project the polarizability tensor of the whole system onto maximally localized Wannier functions (MLWFs)[6]:

$$\alpha = \sum_{i=1}^{N_{\mathrm{orb}}} \alpha_i^{\mathrm{eff}}, \tag{8}$$

where α_i^{eff} is the effective polarizability of the ith Wannier function. The MLWFs, $\{|w_i\rangle\}$, are obtained by a unitary transformation U of the occupied Kohn-Sham orbitals:

$$|w_i\rangle = \sum_{v=1}^{N_{\mathrm{orb}}} U_{iv} |\varphi_v\rangle \tag{9}$$

Using the same unitary matrix, we can also obtain the variation of the MLWFs [7, 8]:

$$|\Delta w_i\rangle = \sum_{v=1}^{N_{\mathrm{orb}}} U_{iv} |\Delta \varphi_v\rangle \tag{10}$$

The polarization electron density of the ith MLWF is

$$\Delta \rho_i = 2 w_i^*(\mathbf{r}) \Delta w_i(\mathbf{r}) + c.c. \tag{11}$$

If we substitute ρ_i into ρ in Supplementary Equation (5) and (6), we obtain α_i^{eff}. The effective polarizability of a molecule or ion is given by:

$$\alpha_{\mathrm{mol}}^{\mathrm{eff}} = \sum_{k=1}^{M} \alpha_k^{\mathrm{eff}} \tag{12}$$

where we sum over the M effective polarizabilities of the MLWFs belonging to a given molecule or ion. Note that the effective polarizability of a molecule or ion is not the *true* molecular or ionic polarizability, because we only consider the macroscopic field $\mathbf{M}$, not the local electric field [9]. The total polarizability of the system can be written as the sum of the effective polarizabilities of molecules and ions in the system:

$$\alpha = \sum_{m=1}^{N_{\mathrm{mol}}} \alpha_{\mathrm{mol},m}^{\mathrm{eff}}, \tag{13}$$

Inter- and intra-molecular Raman spectra

Using the effective molecular polarizability defined in Supplementary Equation (12), we can decompose calculated isotropic Raman spectra as [7],

$$\begin{aligned} R_{\mathrm{iso}}(\omega) &\propto \frac{\hbar\omega}{k_B T} \int dt e^{-\mathrm{i}\omega t} \langle \bar{\alpha}(0)\bar{\alpha}(t) \rangle \\ &= \frac{\hbar\omega}{k_B T} \int dt e^{-\mathrm{i}\omega t} \langle \sum_{\substack{m=1 \\ m \neq n}}^{N_{\mathrm{mol}}} \sum_{n=1}^{N_{\mathrm{mol}}} \bar{\alpha}_{\mathrm{mol},m}^{\mathrm{eff}}(0) \bar{\alpha}_{\mathrm{mol},n}^{\mathrm{eff}}(t) + \sum_{m=1}^{N_{\mathrm{mol}}} \bar{\alpha}_{\mathrm{mol},m}^{\mathrm{eff}}(0) \bar{\alpha}_{\mathrm{mol},m}^{\mathrm{eff}}(t) \rangle \end{aligned} \tag{14}$$

where the first and second terms give the inter- and intra-molecular Raman spectra, respectively. We can also decompose calculated anisotropic Raman spectra in a similar way.

Convergence of the MD simulations

The convergence of our simulations is shown in Fig. 1. When the simulation length is more than 0.6 times the total trajectory time, the peak intensities change by less than 10% and such variation does not affect any of our conclusions.

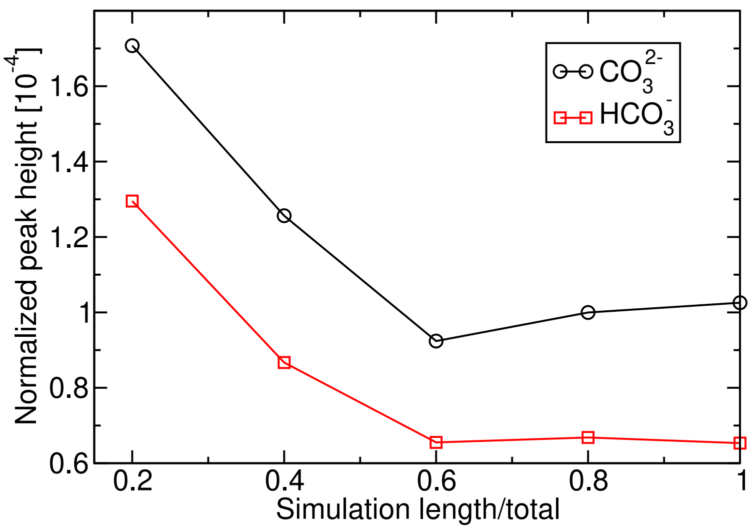

FIG. 1. Convergence of calculated Raman peak intensities. The (bi)carbonate peak intensity in Raman spectra at ambient density and 380 K is shown as a function of the simulation length. The peak intensities are normalized to the water stretching band.

---

* dingpan@ust.hk

[1] Kohn, W. & Sham, L. J. Self-consistent equations including exchange and correlation effects. *Phys. Rev.* **140**, A1133 (1965).

[2] Baroni, S., De Gironcoli, S., Dal Corso, A. & Giannozzi, P. Phonons and related crystal properties from density-functional perturbation theory. *Rev. Mod. Phys.* **73**, 515 (2001).

[3] Sternheimer, R. M. Electronic polarizabilities of ions from the hartree-fock wave functions. *Phys. Rev.* **96**, 951 (1954).

[4] Mahan, G. D. Modified sternheimer equation for polarizability. *Phys. Rev. A* **22**, 1780 (1980).

[5] Baroni, S. & Resta, R. Ab initio calculation of the macroscopic dielectric constant in silicon. *Phys. Rev. B* **33**, 7017–7021 (1986).

[6] Marzari, N., Mostofi, A. A., Yates, J. R., Souza, I. & Vanderbilt, D. Maximally localized wannier functions: Theory and applications. *Rev. Mod. Phys.* **84**, 1419 (2012).

[7] Wan, Q., Spanu, L., Galli, G. A. & Gygi, F. Raman spectra of liquid water from ab initio molecular dynamics: vibrational signatures of charge fluctuations in the hydrogen bond network. *J. Chem. Theory Comput.* **9**, 4124–4130 (2013).

[8] Ge, X. & Lu, D. Local representation of the electronic dielectric response function. *Phys. Rev. B* **92**, 241107 (2015).

[9] Pan, D., Govoni, M. & Galli, G. Communication: Dielectric properties of condensed systems composed of fragments. *J. Chem. Phys.* **149**, 051101 (2018).
